# Prospective Memory, Sleep, and Age

**DOI:** 10.3390/brainsci10070422

**Published:** 2020-07-03

**Authors:** Miranda Occhionero, Lorenzo Tonetti, Marco Fabbri, Michele Boreggiani, Monica Martoni, Sara Giovagnoli, Vincenzo Natale

**Affiliations:** 1 Department of Psychology, University of Bologna, 40127 Bologna, Italy; miranda.occhionero@unibo.it (M.O.); michele.boreggiani@gmail.com (M.B.); sara.giovagnoli@unibo.it (S.G.); vincenzo.natale@unibo.it (V.N.); 2 Department of Psychology, University of Campania Luigi Vanvitelli, 81100 Caserta, Italy; marco.fabbri@unicampania.it; 3 Department of Experimental, Diagnostic and Specialty Medicine, University of Bologna, 40127 Bologna, Italy; monica.martoni@unibo.it

**Keywords:** prospective memory, activity-based task, sleep, age, actigraphy

## Abstract

It is reported that sleep enhances prospective memory (PM), but it remains to be understood whether this influence is moderated by age, since sleep changes across the lifespan. To this end, we performed a retrospective study in a naturalistic setting in a large life span sample: 397 healthy participants (227 females) from middle childhood (nine years old) to late adulthood (70 years old). Participants were requested to perform a naturalistic activity-based PM task, namely, to remember to press the event-marker button of an actigraph when they went to bed (activity 1) and when they got out of bed (activity 2) after nocturnal sleep. The percentages of button presses were the measure of our activity-based PM task. For activities 1 and 2, we separately performed a moderation model with actigraphic sleep parameters (sleep efficiency, midpoint of sleep, and total sleep time) as predictors of PM performance with age as a moderator factor. With reference to activity 1, we observed a significant interaction between sleep efficiency and age, showing a decrease in PM performance with the increase in sleep efficiency in the low age group. Only age was a significant (negative) predictor of PM in activity 2, i.e., with increasing age, PM performance significantly decreased. The present study shows, in a large life span sample, that sleep does not seem to play a relevant predictive role of activity-based PM performance.

## 1. Introduction

Prospective memory (or memory of the future, PM) coordinates, controls, and directs cognitive resources for intentions and future actions [1]. The optimal functioning of PM is crucial in everyday life, as it allows us to successfully complete a wide range of daily activities (family life management, social activities, health care; e.g., Reference [2]). The importance of this memory is also evident from the numerous examples of the potential negative consequences of forgetting to carry out an intention that can be fundamental for our own life (a patient forgets to take a drug, a surgeon does not remember to remove a clamp, an airplane pilot does not adjust the position of the wings before take-off, etc.) [3]. PM also influences many areas of social life. If we arrive late for an appointment, we are hardly believed to be amnesic; it is more likely for us to be considered impolite. For this reason, this type of memory also affects self-esteem, since it is able to influence the perception of oneself as a well-organized or unreliable individual [4]. According to Kvavilashvili and colleagues [5], failure of PM represents 50–70% of all everyday memory problems in the non-clinical population of adults.

Classically, we distinguish two components in literature: a prospective component that elaborates the intention (remember that something must be done) and a retrospective component that involves the contents, i.e., what must be done. These are two interconnected but functionally distinct components [6,7,8,9]. In addition to retrospective memory, there are other cognitive functions involved in PM, including executive functions, working memory, and attentional processes [10,11]. In particular, two processes play a central role in remembering to realize delayed intentions: top-down and bottom-up mechanisms, involved respectively in strategic monitoring and spontaneous recovery. Strategic monitoring (top-down) allows us to keep the intention available in memory and, at the same time, monitors the environment to detect prospective stimuli associated with the intention to be performed. Spontaneous recovery (bottom-up) is involved when a stimulus spontaneously triggers the recovery of intention. These processes were theorized as being part of the Multiprocess Framework Model developed by McDaniel and Eistein [12,13]. Recently, a new model, called the Dynamic Multiprocess Framework, was proposed by Scullin and colleagues [14], according to which strategic monitoring and spontaneous recovery could be used in the same prospective task, but at different times and in distinct contexts.

Based on the characteristics of the intention, Einstein and McDaniel [11] (see also Reference [15]) first distinguished event-based and time-based PM tasks. Event-based tasks mean remembering to perform an intention linked to a specific event, for example, remembering to refuel when you see a gas station along the road. This type of PM task requires an external cue that activates the associated intention to complete the action. Time-based tasks, on the other hand, refer to intentional actions that must be performed at a specific time, for example, remembering to take a drug at 8:00 p.m.

The two types of task differ substantially because they rely upon the presence/absence of an external event or environmental support for retrieval. Time-based tasks depend on self-generated retrieval processes [16], while event-based tasks have external cues that can guide the act of remembering [17]. The event-based tasks can require bottom-up mechanisms while time-based tasks rely on top-down mechanisms because no external cues are available and time monitoring is a self-initiated mental process.

This distinction was subsequently better specified by inserting another category of PM: the so-called activity-based task [18], the most understudied type of PM task. The activity-based task refers to an intention that must be performed before or after a certain activity and, therefore, does not interfere with an ongoing activity. From this point of view, it seems strange that this type of task was little studied since the way we organize our actions most frequently is activity-based. In everyday life, we plan an action following a serial order that configures our daily routine. In addition to seriality in the formulation of intentions, the salience and the cognitive load occupied by an intention play a decisive role. The importance of an intention is the result of a subjective evaluation, based on importance, wishes, and goals [18,19]. According to Walter and Meier [4], the more the intention is considered important, the greater the likelihood of it being remembered, because the capacity of the PM is expanded by the relevance of the intention. Moreover, when a goal is established to perform an activity at the conclusion of a different activity, successful completion suffers compared to when the same intention is associated with a concrete environmental event. Activity-based PM requires a top-down and bottom-up mixed activation. The environmental cue (bottom-up activation) must be integrated with temporal monitoring (top-down activation), which requires an internal and self-generated mechanism of remembering (before going to work I have to take my child to the nursery; after the lesson, I have to make a phone call).

In recent years, the association between sleep and PM was the research focus of both cognitive and sleep scientists, as shown by a recent systematic review and meta-analysis published on this topic [20]. The authors included 20 studies in the meta-analysis, which were published between 2002 and 2019. When examining sleep effects on PM performance, regardless of strategic monitoring and spontaneous recovery, a small-to-medium positive overall effect was observed. However, when they examined strategic monitoring and spontaneous recovery separately, the authors failed to detect a significant sleep effect on strategic monitoring. On the contrary, they observed a sleep-facilitating effect on spontaneous retrieval processes. This pattern of results points out that the topic of the relationship between sleep and PM is still under discussion, as recently demonstrated by Böhm and colleagues [21], who failed to detect a significant association between sleep and PM. Within the research agenda of their study, Leong et al. [20] wrote that “while slow-wave sleep plays a key role in PM in young adults, this relationship may change with age. These age-related changes should be addressed in future studies involving diverse age groups” (page 26).

Keeping this suggestion in mind, we chose to carry out a retrospective study aimed at exploring the association between sleep and PM in a naturalistic setting in a large life span sample, from middle childhood (nine years old) to late adulthood (70 years old). More in detail, we aimed to check whether age can moderate the relationship between sleep and PM. We chose to investigate PM through a naturalistic activity-based task, originally introduced by our research group [22,23] and then also used by others [24,25]. More specifically, participants were originally enrolled in studies in which they were requested to wear an actigraph (a small device able to directly record motor activity data and indirectly monitor sleep) around the non-dominant wrist and to remember to push the event-marker button on the top of actigraph at bedtime (activity 1) and at get-up time (activity 2).

Within a chronobiological perspective, we chose to analyze three actigraphic parameters that correspond to sleep quality (sleep efficiency, SE), timing (midpoint of sleep, MPS), and quantity (total sleep time, TST). These parameters allow us to obtain an overall picture of the potential sleep effects on PM because they mostly change across the lifespan [26,27].

We reasoned that, if sleep influences PM, we would find a larger effect on PM performance after nocturnal sleep, i.e., in activity 2 in comparison to activity 1. This aspect would reflect the so-called sleep effect [28], i.e., better performance when sleep occurs between learning and recall compared to a condition in which sleep is not present during the retention interval. A sleep effect was also previously reported with reference to PM (e.g., Reference [29]). Since sleep architecture varies considerably in different ages, finding a stable relationship between sleep and PM means that sleep per se is a factor linked to prospective performance regardless of the physiological variations that modify its structural characteristics over the lifespan. In particular, we would expect a positive relationship between SE and TST on one hand and PM performance in activity 2 on the other. Taking into account the fact that morning types are more active in the morning [30], we hypothesized that morningness could facilitate performance in the morning; therefore, a negative association between MPS and PM performance in activity 2 could be expected. Finally, we did not expect any significant sleep effect on PM performance in activity 1, as we assumed that this activity would be modulated mostly by diurnal factors.

## 2. Materials and Methods

### 2.1. Participants

**Figure 1:**
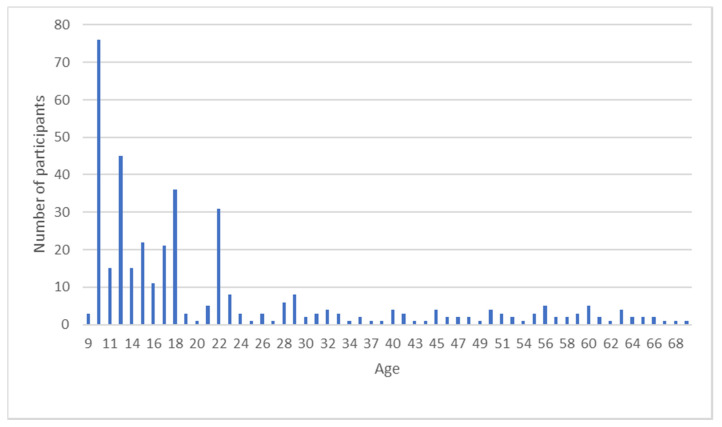
Frequency distribution of age.

In this study, the participants came from different previous experiments [31,32,33,34,35,36]. The complete sample consisted of 397 healthy participants (170 males, 227 females) with an age ranging from nine to 70 years (mean age: 22.79 ± 15.56); the frequency distribution of age is shown in Figure 1. The participants were part of a large sample of people with regular sleep habits, with no drug or medication use that may affect sleep and/or cognition.

Sleep–wake cycle was monitored using the Actiwatch AW64 actigraph (Cambridge Neurotechnology Ltd., Cambridge, UK) for a total of 2926 nights.

### 2.2. Materials and Procedure

During data collection, all participants maintained a regular sleep–wake cycle, monitored by the Actiwatch AW64 actigraph (Cambridge Neurotechnology Ltd., Cambridge, UK). This instrument is a small device that measures motor activity during a 24-h period; it is especially useful for assessing the individual sleep–wake cycle at home (naturalistic setting) for multiple nights when no sleep stage identification is required [37].

The hardware consists of a piezoelectric accelerometer with a sensitivity of ≥0.05 g. The sampling frequency was 32 Hz, and filters were set to 3–11 Hz. Actigraphs were initialized by Actiwatch Activity and Sleep Analysis 5 software (version 5.32, Cambridge Neurotechnology Ltd.; Cambridge, UK) to collect data in 1-min epochs. The same software analyzed actigraph data files. The participants wore the actigraph on their non-dominant wrist for a mean period of 7.37 ± 2.40 consecutive days and simultaneously compiled a sleep log within 30 min of waking up.

At the beginning of the experimental period, participants were instructed to press the event-marker button of the actigraph when they went to bed (bedtime) and got out of bed (get-up time). The percentages of button presses were the measure of our naturalistic activity-based PM task. The instructions were not repeated during the recording period. Being in one’s bedroom and the actions of getting in and out of bed were the only reminders of the task [11] at bedtime (activity 1) and upon morning awakening (activity 2). At the same time, actigraphic recordings provided quantitative data of individuals’ sleep. Sleep quality, timing, and quantity were examined using the following sleep parameters:

- SE: the percentage of total sleep time compared to time spent in bed.
- MPS: the clock time that splits the interval between sleep start and sleep end in half.
- TST: minutes of sleep during the assumed sleep period.

For activities 1 and 2, the activity-based PM performance was separately scored as correct (1: the intention was executed at bedtime or get-up time) or incorrect (0: the intention was not executed at all). For each participant, we computed the percentages of correct execution of the PM task for both activities because the total number of button-presses varied from participant to participant based on how many days the actigraph was worn.

### 2.3. Statistical Analysis

All statistical analyses were performed using SPSS statistical package (version 25; IBM Corp., Armonk, NY, USA). A set of generalized linear models was used to study the association between the variables of interest in this study. The generalized linear model assumes that the dependent variable is linearly related to the factors and covariates via a specified link function. Moreover, the model allows for the dependent variable to have a non-normal distribution, as in the case of the variables used in this research. The statistical assumption of the generalized linear model (statistical independence of observations, correct specification of link function) was carefully checked and criteria were met.

Preliminary analyses were conducted to assess the effect of gender and the effect of the variable “number of nights recorded” on PM performance (both for activity 1 and for activity 2). No differences were found between males and females either for PM activity 1 (Wald chi-square = 1.314; *p* = 0.25) or for PM activity 2 (Wald chi-square = 2.164; *p* = 0.14). Not even the number of nights recorded showed a significant effect on PM performance (activity 1: Wald chi-square = 0.385, *p* = 0.54; activity 2: Wald chi-square = 0.062; *p* = 0.80). Accordingly, the variables gender and number of nights recorded were not included in the subsequent statistical analyses.

To evaluate the effect of age on sleep quality (SE), quantity (TST), and timing (MPS), three linear regression analyses were applied separately by means of the generalized linear models. In each model, age was the predictor variable, and the sleep parameters were used as dependent variables.

In order to detect any associations between age, sleep, and activity-based PM, we decided to perform two moderation models using the generalized linear model in which we set all actigraphic sleep parameters (SE, TST, MPS) as predictors of PM performance using age as moderator factor. To perform the moderation models by means of the generalized linear model, the main effects of the sleep parameters and age, and the interaction effects of age with each sleep parameter (moderation effect) were analyzed.

## 3. Results

### 3.1. Age and Sleep

**Figure 2:**
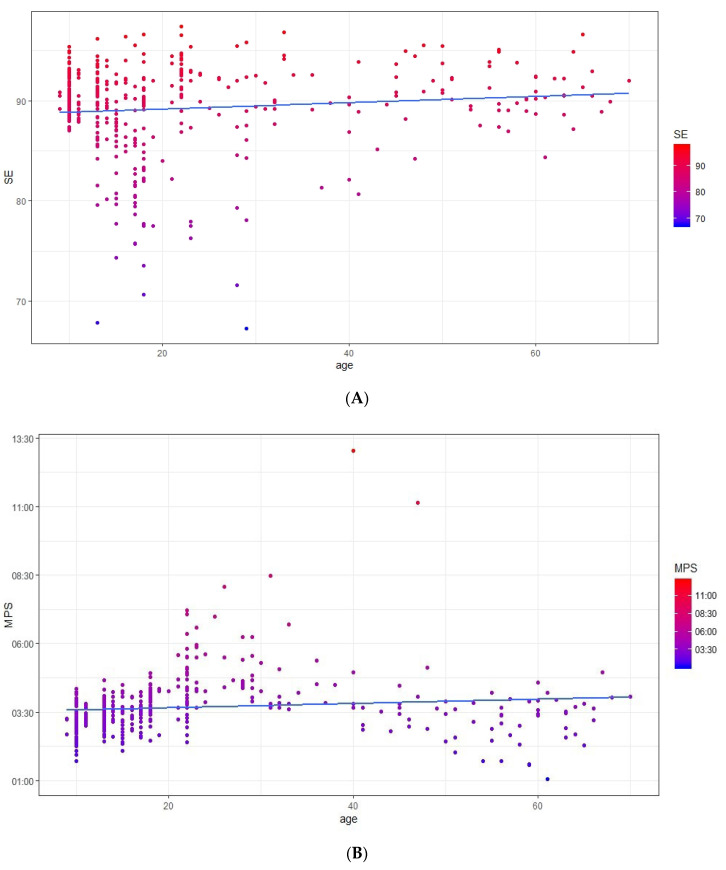
Scatterplots showing the relationship between age and the sleep efficiency (SE) (%) sleep parameter (**A**) and the relationship between age and the midpoint of sleep (MPS) (clock time) sleep parameter (**B**).

All three generalized linear models produced significant levels (SE: Wald chi-square = 4.131, *p* < 0.05; MPS: Wald chi-square = 4.120, *p* < 0.05; TST: Wald chi-square = 17.527, *p* < 0.001), attesting that age was predictive of sleep parameters. As Figure 2 shows, age positively predicted the SE (Figure 2A) and MPS (Figure 2B) parameters; in detail, with increasing age, a significant increase in the SE parameter was observed (b = 0.005, 95% confidence intervals: 0.000; 0.011). The same relationship was observed for the MPS parameter (b = 0.418, 95% CI: 0.014; 0.821), with a delay of the MPS with aging. The whole sample showed a mean SE equal to 89.22 (SD = 4.66), while the average MPS was 03:40 (SD = 1:07).

**Figure 3:**
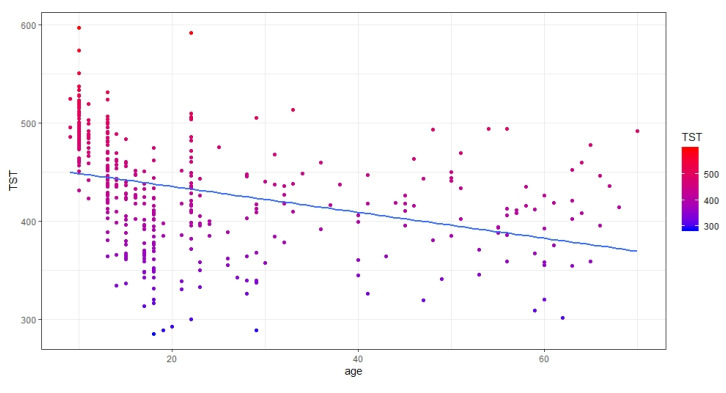
Scatterplot showing the relationship between age and the total sleep time (TST) (minutes) sleep parameter.

On the contrary, a significant negative relationship was found between age and TST. Results show that, as age increases, the TST parameter significantly decreases (b = −0.273, 95% CI: −0.400; −0.145) (Figure 3). The mean TST found was equal to 431.92 (SD = 57.86).

### 3.2. The Moderation Effect of Age on the Relationship Between Sleep and PM Performance

#### 3.2.1. Relationship between Sleep and PM Performance in Activity 1: The Moderator Role of Age

Bearing in mind that the dependent variable (PM activity 1) was not normally distributed, in order to evaluate the moderator role of age in the relationship between sleep parameters and PM performance in activity 1, we executed a linear regression analysis by means of the generalized linear model. The whole sample showed a mean PM performance in activity 1 equal to 82.6% (SD = 20.2).

Results showed a significant SE by age interaction effect (moderator effect: Wald chi-square = 5.24, *p* < 0.05), attesting that SE resulted to be predictive for PM performance in activity 1 when moderated by age. As Table 1 shows, no other significant effects were found in the moderator model.

**Figure 4:**
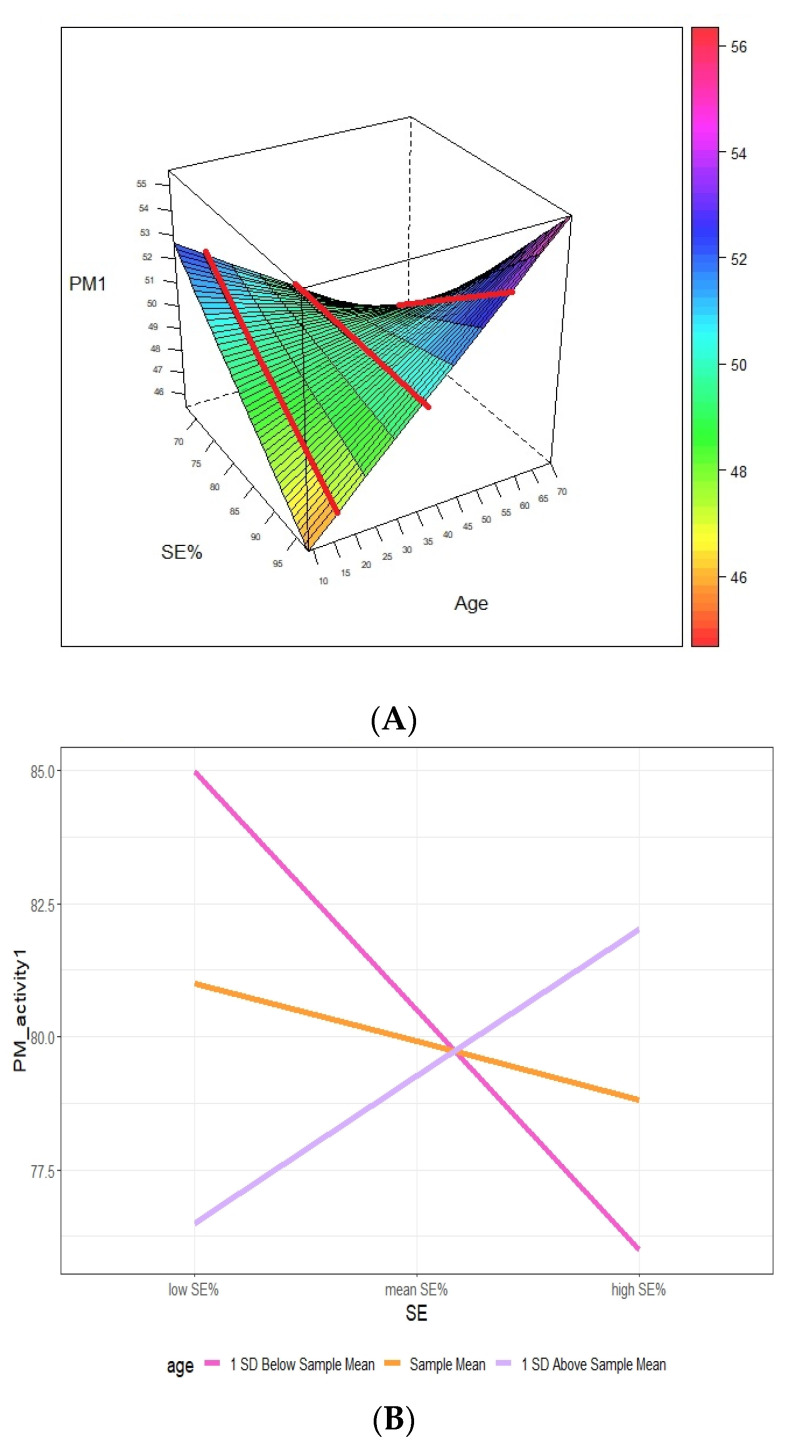
The relationship between SE (%) and PM activity 1 (%) at high, average, and low levels of age. (**A**) A three-dimensional (3D) graph of the relationship; (**B**) classical scatterplot of the relationship between variables. When the reference lines (red lines) drawn in Figure 4A are taken into account and rotated clockwise, they overlap, forming the configuration shown in Figure 4B.

Simple slope analyses were conducted to explore the nature of the two-way interactions between SE and age. Based on the suggestions by Aiken and West [38], the simple slope was obtained by calculating predicted values of activity 1 under different SE and age conditions. Calculation of values corresponding to the mean, one standard deviation above and below the mean, for both predictor and moderator, was used to plot the variables and to test the statistical significance for each of the simple slopes. The predicted relationship between activity 1 and SE at different levels of age (i.e., −1 SD, mean, and +1 SD) is shown in Figure 4A,B.

The results indicate that the relationship between SE and PM activity 1 was negative and significant in the low age group (one standard deviation below the mean; b = 0.965, t = −2.347, *p* < 0.05), but was not statistically significant in the high age group (one standard deviation above the mean; b = 0.591, t = 1.177, *p* = 0.24) or in the mean age group (b = −0.234, t = −0.890, *p* = 0.37). In detail, in the low age group, PM performance in activity 1 decreased with the increase in SE parameter.

#### 3.2.2. Relationship between Sleep and PM Performance in Activity 2: The Moderator Role of Age

A second linear regression analysis by means of the generalized linear model was applied to evaluate the moderator role of age in the relationship between sleep parameters and PM performance in activity 2.

**Figure 5:**
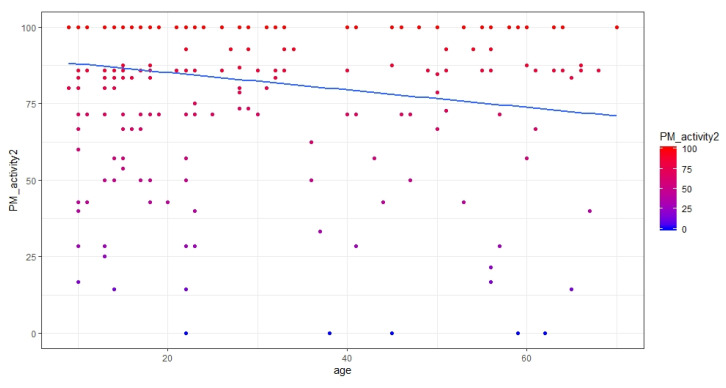
Scatterplot showing the relationship between age and PM performance in activity 2 (%).

Only age resulted significantly predictive of PM activity 2 (b = −0.146, chi-square = 4.240, *p* < 0.05). The relationship was negative, pointing out that, with increasing age, performance in activity 2 significantly decreased. Figure 5 shows the relationship between age and PM activity 2.

As Table 2 shows, neither significant main effects nor significant interaction effects were found. The results show that sleep parameters are not significant predictors of PM activity 2, even when the relationship between sleep and PM activity 2 is moderated by age. The whole sample showed a mean PM performance in activity 2 equal to 86.6% (SD = 19.9).

## 4. Discussion

The aim of the present study was to examine in depth the relationship between sleep and PM in a large life span sample, one that ranged from middle childhood to late adulthood. In line with a suggestion put forward by Leong et al. [20], we were mainly interested in understanding whether age can moderate such an association. Moreover, if nocturnal sleep influences PM performance, we expected to find a larger effect for activity 2, i.e., get-up time.

With reference to the relationship between age and sleep, aging is associated with an increase in SE (a marker of sleep quality, Figure 2A), a delay of MPS (a marker of sleep timing, Figure 2B), and a decrease in TST (a marker of sleep quantity, Figure 3). To briefly discuss such results, we reference the meta-analytic study by Ohayon and colleagues [26] that investigated age-related sleep changes during the life span by examining both polysomnographic and actigraphic studies. Our results are partially in line with those reported by Ohayon and colleagues [26]; indeed, we confirmed a decrease in TST with increasing age. However, while Ohayon et al. [26] reported a decrease in SE with aging, we disclose the opposite relationship, albeit with a very small b-value, i.e., 0.005. Moreover, when discussing these results, we must bear in mind that, although we investigated a large life span sample, most of our participants were aged under 30 years old (Figure 1).

With regard to PM performance in activity 1, i.e., bedtime, we observed a significant interaction between SE and age (Table 1), showing, in the low age group, a decrease in PM performance with the increase in SE (Figure 4). A potential explanation for this finding is related to the homeostatic sleep regulation process [39], the main marker of which is slow-wave activity during NREM sleep. Since slow-wave sleep is mostly prevalent at an early stage of life [26], it is possible to suggest that a higher homeostatic sleep pressure, which decreases alertness levels, may have interfered with the execution of the activity-based PM task at bedtime. However, we must acknowledge that this is a mere speculative hypothesis that we are not able to verify due to the limit of the lack of any information about sleep architecture.

With reference to PM performance in activity 2, no main effects of any actigraphic sleep parameters were observed, nor were there any interactions between any of them and age (Table 2). The only main effect observed was that of age (Table 2), with a decrease in PM performance with increasing age (Figure 5). These data confirm the hypothesis that, with advancing age, even prospective memory, in the same way as for retrospective memory [12,40,41], may undergo a process of impairment that is probably due to a reduced efficiency of attentional processes [42]. This pattern of results would seem to point out that sleep quality, quantity, and timing are not strongly related to PM after nocturnal sleep, at least in this specific task, i.e., activity-based, carried out in a naturalistic setting. This result could be interpreted within the framework of the relationship between sleep and PM as recently described by Leong et al. [20] in their meta-analytic study. They observed a small effect size for event-based tasks, in part similar to the activity-based task used in the present study, as well as a small and non-significant effect in older people; there were also some older people in our sample, although they were few. The negative predictive role played by age in PM performance is in line with recent evidence that the consolidation of delayed intention in PM undergoes a process of deterioration with aging, which could be, at least partially, mediated by the decrease in rapid eye movement (REM) sleep with increasing age [43].

While some discrepancies were detected between PM performance in activity 1 and activity 2, the main take home message of the present study is that a sleep effect on PM, examined in a naturalistic setting in a large life span sample, was not observed.

Some limitations of the present study should necessarily be disclosed. Firstly, older age participants were underrepresented in the sample (Figure 1). Secondly, we used just one type of PM task. Thirdly, actigraphy did not allow us to assess sleep architecture.

However, our study also presents some strengths. Firstly, the sample size was large, at least for an actigraphic study. Secondly, the use of the actigraphic technique allowed for an objective monitoring of sleep timing, quality, and quantity. Thirdly, the PM task was performed in a naturalistic setting, increasing the ecological validity of our findings.

Future studies should try to explore the moderator role of age in the association between sleep and PM by examining a large life span sample that is balanced among different age groups, as well as by administering different types of PM task.

## 5. Conclusions

The present study examined the relationship between sleep, age, and activity-based PM. The results confirm the importance of age in PM performance while it does not seem that sleep characteristics are strongly related to PM. These results encourage the development of future research with event- and time-based PM tasks in different normal age groups, as well as in clinical populations.

## Figures and Tables

**Table 1:** Parameter estimates of the relationship between sleep parameters and prospective memory (PM) activity 1 moderated by age.

Dep: PM Activity 1 *n* = 386	Predictors	B (SE)	95% Wald CI (LLCI–ULCI)	Wald Chi^2^
Main effect				
	SE	−0.005	−0.088–0.079	0.013
	TST	0.042	−0.063–0.147	0.617
	MPS	−0.050	−0.157–0.057	0.839
	Age	−0.016	−0.109–0.077	0.110
Interaction effect				
	SE by age	0.122	0.004–0.239	4.133 *
	TST by age	−0.047	−0.139–0.045	0.988
	MPS by age	0.078	−0.011–0.167	2.936

SE = sleep efficiency; TST = total sleep time; MPS = midpoint of sleep; B = unstandardized estimated coefficient; LLCI = lower level confidence interval; ULCI = upper level confidence interval. * *p* < 0.05.

**Table 2:** Parameter estimates of the relationship between sleep parameters and PM activity 2 moderated by age.

Dep: PM Activity 2 *n* = 387	Predictors	B (SE)	95% Wald CI (LLCI–ULCI)	Wald Chi^2^
Main effect				
	SE	0.078	−0.038–0.194	1.75
	TST	0.081	−0.045–0.207	1.596
	MPS	0.031	−0.094–0.157	0.24
	Age	−0.146	−0.285–−0.007	4.24 *
Interaction effect				
	SE by age	0.142	−0.026–0.310	0.756
	TST by age	−0.065	−0.168–0.037	0.551
	MPS by age	0.005	−0.121–0.132	0.007

SE = sleep efficiency; TST = total sleep time; MPS = midpoint of sleep; B = unstandardized estimated coefficient; LLCI = lower level confidence interval; ULCI = upper level confidence interval. * *p* < 0.05.
